# Human cancer-associated fibroblasts enhance glutathione levels and antagonize drug-induced prostate cancer cell death

**DOI:** 10.1038/cddis.2017.225

**Published:** 2017-06-01

**Authors:** Emarndeena H Cheteh, Martin Augsten, Helene Rundqvist, Julie Bianchi, Victoria Sarne, Lars Egevad, Vladimir JN Bykov, Arne Östman, Klas G Wiman

**Affiliations:** 1 Department of Oncology-Pathology, Cancer Center Karolinska (CCK), Karolinska Institutet, Stockholm, Sweden; 2 Division for Vascular Oncology and Metastasis, German Cancer Research Center, Heidelberg, Germany; 3 Department of Cell and Molecular Biology, Karolinska Institutet, Stockholm, Sweden

## Abstract

Drug resistance is a major problem in cancer therapy. A growing body of evidence demonstrates that the tumor microenvironment, including cancer-associated fibroblasts (CAFs), can modulate drug sensitivity in tumor cells. We examined the effect of primary human CAFs on p53 induction and cell viability in prostate cancer cells on treatment with chemotherapeutic drugs. Co-culture with prostate CAFs or CAF-conditioned medium attenuated DNA damage and the p53 response to chemotherapeutic drugs and enhanced prostate cancer cell survival. CAF-conditioned medium inhibited the accumulation of doxorubicin, but not taxol, in prostate cancer cells in a manner that was associated with increased cancer cell glutathione levels. A low molecular weight fraction (<3 kDa) of CAF-conditioned medium had the same effect. CAF-conditioned medium also inhibited induction of reactive oxygen species (ROS) in both doxorubicin- and taxol-treated cancer cells. Our findings suggest that CAFs can enhance drug resistance in cancer cells by inhibiting drug accumulation and counteracting drug-induced oxidative stress. This protective mechanism may represent a novel therapeutic target in cancer.

Cancer develops by stepwise acquisition of genetic alterations that endow tumor cells with a set of critical properties, including insensitivity to antigrowth signaling, evasion of apoptosis and ability to migrate and form metastasis.^1, 2^ Tumors can be regarded as complex organs composed of tumor cells and a variety of nonmalignant stromal cells that form the tumor microenvironment. These stromal cells include endothelial cells, pericytes, immune inflammatory cells and cancer-associated fibroblasts (CAFs), all of which presumably have an important role during tumorigenesis.^2, 3^ These cells are relatively genetically stable and are typically not malignantly transformed. However, they are influenced by the interaction with tumor cells and display altered gene expression patterns that favor tumor development, tumor growth and invasion.^4, 5^ Several of the affected genes encode secreted and cell surface proteins. It is known that the tumor microenvironment can interact with tumor cells through soluble proteins, such as cytokines and growth factors, that mediate paracrine or juxtacrine signaling.^6^

CAFs are among the most crucial components in the prostate tumor microenvironment and are important modulators of prostate tumorigenesis.^7^ Several *in vitro* and *in vivo* studies have demonstrated that prostate cancer-derived CAFs are able to transform nontumorigenic prostate epithelial cells,^8, 9^ and affect the proliferation or the invasiveness of the cancer cells.^10, 11^ CAFs are also important producers of growth factors, cytokines or extracellular matrix proteins, some of which have important roles in cancer drug resistance. A recent study demonstrated that prostatic CAFs can influence the response of prostate cancer cells to androgens and anti-androgens.^12^ Another study found that prostatic CAFs secrete WNT16B following chemotherapy, which increases cancer cell drug resistance *in vitro* and *in vivo*.^13^ CAFs have also been shown to protect colorectal cancer cells from oxaliplatin and 5-FU.^14^

The tumor suppressor p53 has a critical role as guardian against tumor development. Wild-type p53 is implicated in a wide range of cellular processes and responses such as growth arrest, DNA repair, metabolism and apoptosis by differentially regulating target gene expression. p53 is rapidly induced by various types of stress, for instance DNA damage or oncogene activation. The TP53 gene is frequently mutated in human tumors.^15^ The frequency of TP53 mutations in prostate cancer is relatively low, 5–25%, but it is higher in metastatic lesions according to some studies.^16, 17, 18^ In general, tumors that carry mutant TP53 are more resistant to chemotherapy and radiotherapy, consistent with the notion that DNA-damaging drugs and radiotherapy induce tumor cell death at least in part through the activation of wild-type p53.

Chemotherapeutic agents such as mitomycin C, taxol and doxorubicin have long been used to treat solid tumors, but development of drug resistance remains a substantial problem. Former studies on drug resistance have mainly focused on cancer cells themselves. Here we have investigated how CAFs might influence sensitivity of prostate cancer cells to chemotherapeutic agents. We demonstrate that CAFs inhibit drug-induced cell death in wild-type TP53-carrying prostate cancer cells. We also show that CAFs enhance prostate cancer cell glutathione levels, inhibit drug-induced reactive oxygen species (ROS) and doxorubicin accumulation.

## Results

### CAFs and CAF-conditioned medium increase cancer cell survival in response to chemotherapeutic drugs

To investigate the effect of prostatic fibroblasts on the survival of prostate cancer cells on treatment with chemotherapeutic drugs, wild-type TP53-carrying LNCaP prostate cancer cells were co-cultured in a transwell system under physical separation with cancer-associated fibroblasts (CAFs) or normal fibroblasts (NFs) from patients (cancer cell-fibroblast ratio 2:1). We selected LNCaP cells for our studies as a representative example of wild-type TP53-carrying prostate cancer cells. The CAFs used in our studies were established from prostates of different prostate cancer patients and pooled together to minimize excessive data spread due to interindividual variation. NFs were derived from morphologically normal regions of prostates from the same patients.

**Figure 1:**
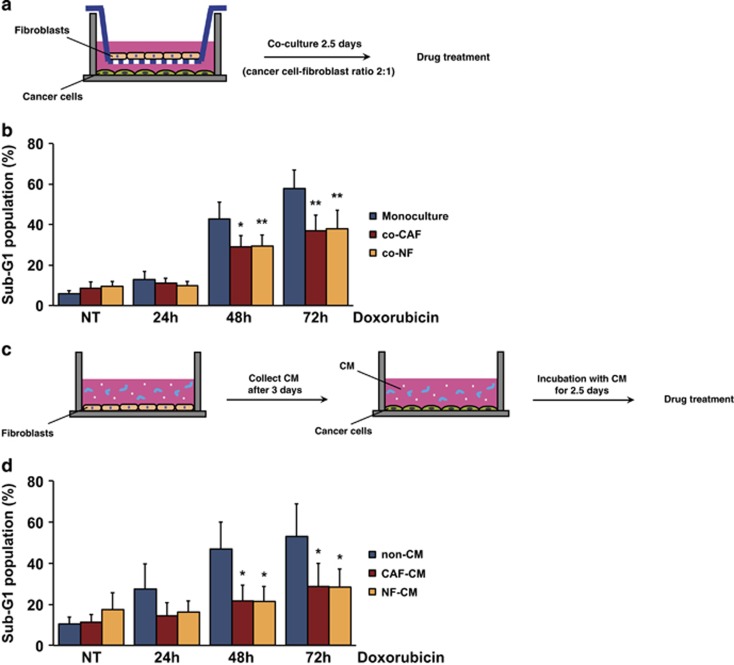
CAFs inhibit drug-induced LNCaP cell death. (**a**) Schematic of the experimental set-up for transwell co-culture assay. (**b**) FACS-PI analysis showing sub-G1 cell population of LNCaP cells co-cultured with fibroblasts (CAF or NF) after 24, 48 and 72 h of 1 *μ*M doxorubicin treatment (mean and S.E.M.; *N*=7; *P<0.05, **P<0.01). (**c**) Schematic image of the conditioned medium (CM) assay. (**d**) Sub-G1 cell population of LNCaP cells grown with fresh (non-CM) or fibroblast- (CAF or NF) conditioned medium (CM), as determined by FACS-PI analysis after 24, 48 and 72 h exposure of 1 *μ*M doxorubicin (mean and S.E.M.; *N*=5; *P<0.05). NT, non-treated

Following culture of LNCaP and primary prostate fibroblasts (CAFs or NFs) in the transwell chamber for 2 to 3 days, the cells were treated with chemotherapeutic drugs (Figure 1a) and LNCaP cell viability was assessed at various time points. Sub-G1 cell population indicative of cell death was assessed by flow cytometry. LNCaP cells cultured with CAFs showed reduced cell death upon 48 and 72 h treatment with doxorubicin (Figure 1b), taxol (Supplementary Figure 1A) and mitomycin C (data not shown), as compared with LNCaP cells in monoculture. NFs showed similar efficacy as CAFs in inhibiting drug-induced cancer cell death (Figure 1b and Supplementary Figure 1A). The sub-G1 population was reduced by 20–30% in co-culture conditions after doxorubicin treatment, and by 10–20% after taxol and mitomycin C treatment at 48 and 72 h. Thus, cell death induced by doxorubicin, taxol and mitomycin C was attenuated by co-culture with the fibroblasts.

In line with these findings, LNCaP cells grown in CAF- or NF-conditioned medium (Figure 1c) also exhibited up to 30% decrease in cell death, as compared with cells grown in non-conditioned medium, after 48 and 72 h of doxorubicin treatment (Figure 1d). This suggests that one or several fibroblast-derived soluble factors protect LNCaP cells from drug-induced cell death.

Human diploid fibroblasts (HDFs), hTERT-immortalized BJ human fibroblasts (BJ hTERT) and LNCaP cells themselves did not show significant protective effect on co-cultured LNCaP cells after 48 h doxorubicin exposure and only little effect at 72 h (Supplementary Figure 1B).

### CAFs and CAF-conditioned medium attenuate p53 induction in prostate cancer cells treated with chemotherapeutic drugs

**Figure 2:**
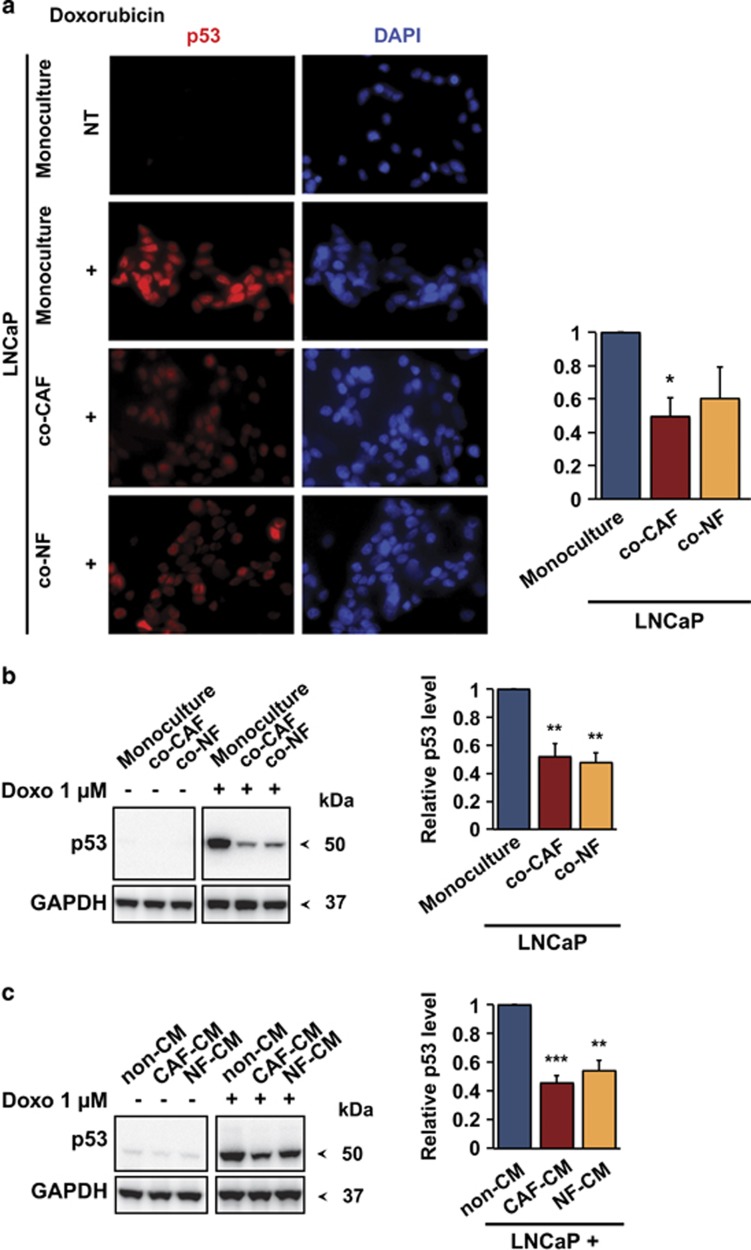
CAFs attenuate the induction of p53 in LNCaP cells. (**a**) Left, representative immunofluorescence staining of p53 (red) in LNCaP cells co-cultured with fibroblasts (CAF or NF) after 9 h of 1 *μ*M doxorubicin treatment. Cell nuclei were stained with DAPI (blue). LNCaP-eGFP cells were used to distinguish LNCaP cells from the fibroblasts. Original magnification × 40. Right, quantitative graph of nuclear p53 staining after doxorubicin treatment in relation to p53 intensity in doxorubicin-treated monoculture (mean and S.E.M.; *N*=3; *P<0.05). NT, non-treated. (**b**) Left panel: p53 accumulation after 9 h of 1 *μ*M doxorubicin treatment in LNCaP cells co-cultured with fibroblasts (CAF or NF) using transwell system, as indicated by a representative immunoblot of whole-cell lysates. Right panel: quantification of p53 level after drug treatment in relation to p53 level of doxorubicin-treated monoculture (mean and S.E.M.; *N*=6; **P<0.01). (**c**) Left panel: p53 accumulation in LNCaP cells after incubation with fresh (non-CM) or (CAF- or NF-) conditioned medium (CM) and 9 h of 1 *μ*M doxorubicin treatment, as indicated by a representative immunoblot. Right panel: quantification of p53 level after the treatment in relation to p53 level of control treated with non-conditioned medium and doxorubicin (mean and S.E.M.; *N*=7; **P<0.01, ***P<0.0001)

To examine whether the effect of fibroblasts on cancer cell death on treatment with chemotherapeutic drugs involved modulation of p53, we exposed LNCaP cells to fibroblasts in co-culture or to fibroblast-conditioned medium and assessed p53 protein levels on treatment with doxorubicin, mitomycin C or taxol. We observed a 50% reduction in p53 immunostaining intensity in doxorubicin-treated LNCaP cells grown in direct-contact co-culture with CAFs or NFs, as compared with LNCaP cells grown alone (Figure 2a). Western blotting also showed that CAFs or NFs attenuated p53 accumulation in LNCaP cells on treatment with doxorubicin (Figure 2b), taxol (Supplementary Figure 2A) and mitomycin C (Supplementary Figure 2B). Similarly, conditioned medium from CAFs or NFs decreased the doxorubicin-induced (Figure 2c) and taxol-induced (Supplementary Figure 2C) p53 accumulation in LNCaP cells. Co-culture with HDFs, BJ hTERT, LNCaP cells or conditioned medium from these cells did not have any significant impact on the doxorubicin-induced p53 response in LNCaP cells (Supplementary Figures 3A and B).

### CAF-conditioned medium decreases DNA damage in prostate cancer cells treated with doxorubicin

**Figure 3:**
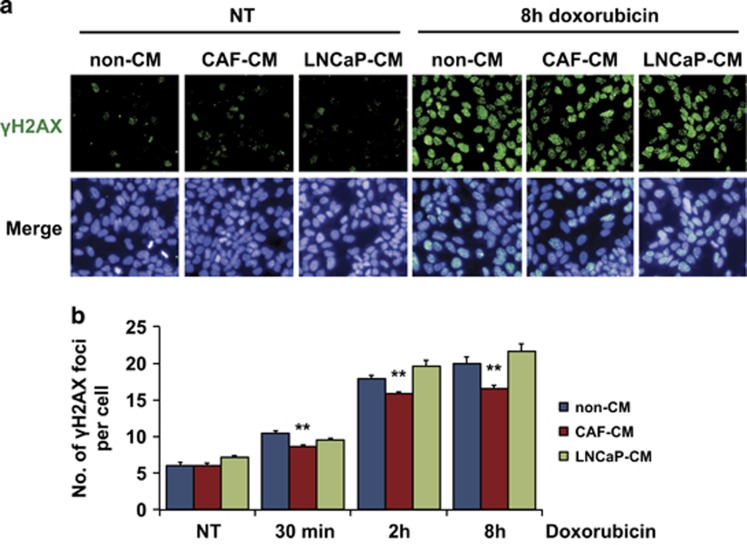
Decreased DNA damage in LNCaP cells cultivated in CAF-CM. (**a**) Representative images of *γ*H2AX foci (green) and nuclei (blue) visualized with Operetta, an automated fluorescence microscope, after 8 h of doxorubicin treatment. Original magnification × 20. (**b**) Average number of *γ*H2AX foci per cell after 30 min, 2 h and 8 h of 1 *μ*M doxorubicin treatment (mean and S.E.M.; *N*=3; **P<0.01). The foci numbers were obtained with Operetta and analyzed with Columbus software

Doxorubicin induces DNA damage by inhibiting topoisomerase II, resulting in DNA double-strand breaks.^19^ Therefore, we examined the levels of doxorubicin-induced DNA damage in LNCaP cells by analyzing phosphorylation of histone H2AX at Ser139 (*γ*H2AX). We assessed the number of *γ*H2AX foci per cell nucleus by the Operetta automated fluorescence microscope system at different time points following doxorubicin treatment. The average number of *γ*H2AX foci increased with the time of drug exposure, and was significantly lower in LNCaP cells grown in CAF-conditioned medium compared with the cells grown in non-conditioned medium or LNCaP-conditioned medium (Figures 3a and b).

Taken together, our results show that LNCaP cells are less sensitive to chemotherapeutic drugs in the presence of CAFs or CAF-conditioned medium, indicating a pivotal role of CAFs in drug resistance.

### CAF-conditioned medium decreases the accumulation of doxorubicin in cancer cells

**Figure 4:**
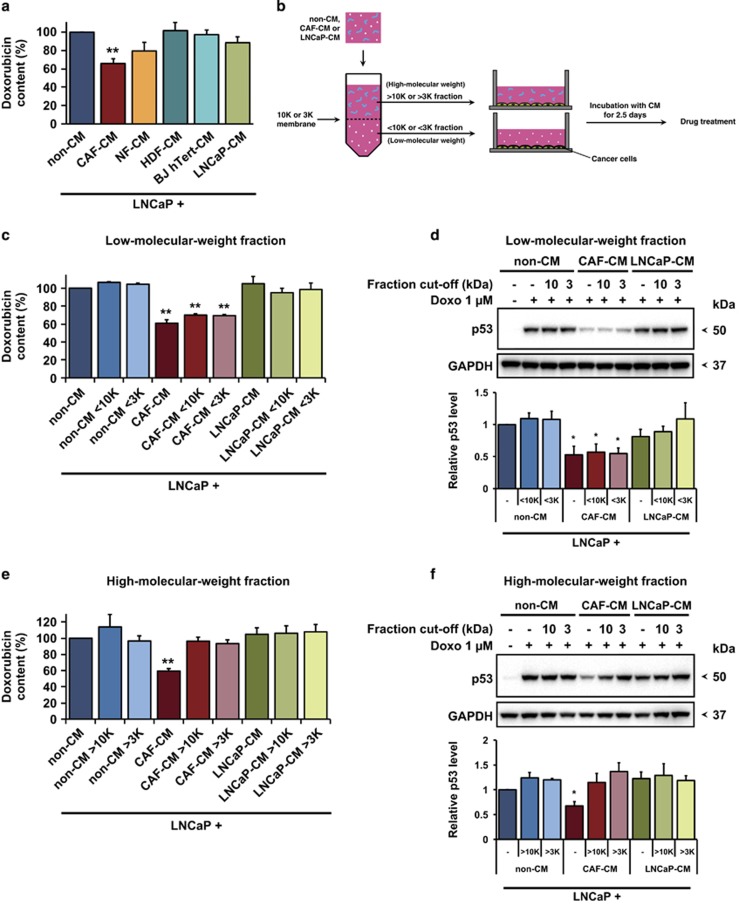
The low molecular weight fraction of CAF-CM decreases doxorubicin accumulation in LNCaP cells. (**a**) Doxorubicin content assessed by FACS in LNCaP cells exposed to various conditioned media (CM) and 8 h of 1 *μ*M doxorubicin (mean and S.E.M.; *N*=5; **P<0.01). (**b**) Schematic diagram of the separation of fresh (non-CM) or CAF- or LNCaP-conditioned medium (CM) into high- and low molecular weight fractions with cut-off of 10 kDa or 3 kDa. (**c**) Effect of low molecular weight fraction on doxorubicin accumulation, as assessed by FACS (mean and S.E.M.; *N*=3; **P<0.01) and (**d**) upper panel: p53 induction in LNCaP cells exposed to 8 h of 1 *μ*M doxorubicin, as indicated by a representative immunoblot. Lower panel: quantification of p53 level after the treatment in relation to p53 level of control treated with non-conditioned medium and doxorubicin (mean and S.E.M.; *N*=4; *P<0.05). (**e**) Effect of high molecular weight fraction on intracellular doxorubicin level as assessed by FACS (mean and S.E.M.; *N*=3; **P<0.01) and (**f**) upper panel: p53 induction in LNCaP cells exposed to 1 *μ*M doxorubicin for 8 h, as shown by a representative immunoblot. Lower panel: quantification of p53 level after the treatment in relation to p53 level of control treated with non-conditioned medium and doxorubicin (mean and S.E.M.; *N*=4; *P<0.05)

Several mechanisms may contribute to drug resistance in cancer, including reduced drug uptake, enhanced drug efflux, increased drug metabolism, augmented repair of damaged DNA and inhibition of cell death.^20^ To examine whether the attenuated p53 induction and cell death response to doxorubicin in LNCaP cells was associated with changes in drug accumulation, we took advantage of the fact that doxorubicin displays a strong fluorescence which allows assessment of intracellular doxorubicin content by flow cytometry.^21, 22^ As shown in Figure 4a, doxorubicin accumulation in LNCaP cells grown in CAF-conditioned medium was decreased by almost 40% as compared to cells treated with doxorubicin in non-conditioned medium. NF-conditioned medium also caused decreased doxorubicin accumulation, but not to the same extent as CAF-conditioned medium. Conditioned medium from HDFs, BJ hTERT fibroblasts and LNCaP cells showed very mild or no effect on doxorubicin accumulation in LNCaP cells.

We obtained similar results with regard to intracellular doxorubicin accumulation and p53 protein levels in 22Rv1 cells, another prostate cancer cell line with functional p53 protein (Supplementary Figures 4A and B).

In contrast, CAF-conditioned medium inhibited taxol-induced cell death without significantly affecting taxol accumulation in LNCaP cells (Supplementary Figure 5). We observed a slightly increased rate of both taxol uptake and efflux in cells cultured with CAF-conditioned medium after 4 h of drug removal.

These findings suggest that CAF-derived factors inhibit doxorubicin accumulation in prostate cancer cells, and reveal one potential mechanism by which CAFs can protect cancer cells from drug-induced cell death.

### The low molecular weight fraction of CAF-conditioned medium decreases doxorubicin accumulation in cancer cells

To identify the CAF-derived factors that mediate drug resistance, we fractionated CAF-conditioned medium using centrifugal filter units with a 10 kDa or 3 kDa membrane cut-off (Figure 4b). The fraction containing components with a molecular weight lower than 3 kDa was able to inhibit intracellular doxorubicin accumulation (Figure 4c) and attenuate p53 protein induction (Figure 4d) with similar efficiency as the unfractionated CAF-conditioned medium. In contrast, the fraction containing components with a molecular weight above 3 kDa did not interfere with doxorubicin accumulation and doxorubicin-induced p53 protein levels (Figures 4e and f). Furthermore, the low molecular weight fraction (<3 kDa) from non-conditioned medium or LNCaP-conditioned medium had very little or no effect on both accumulation of doxorubicin and p53 (Figures 4c and d). As control, all fractions of CAF-conditioned medium were pooled and re-tested on LNCaP cells. The pooled fractions were as potent as the unfiltered CAF-conditioned medium (Supplementary Figures 6A and B).

### Role of glutathione and its precursors for doxorubicin accumulation

**Figure 5:**
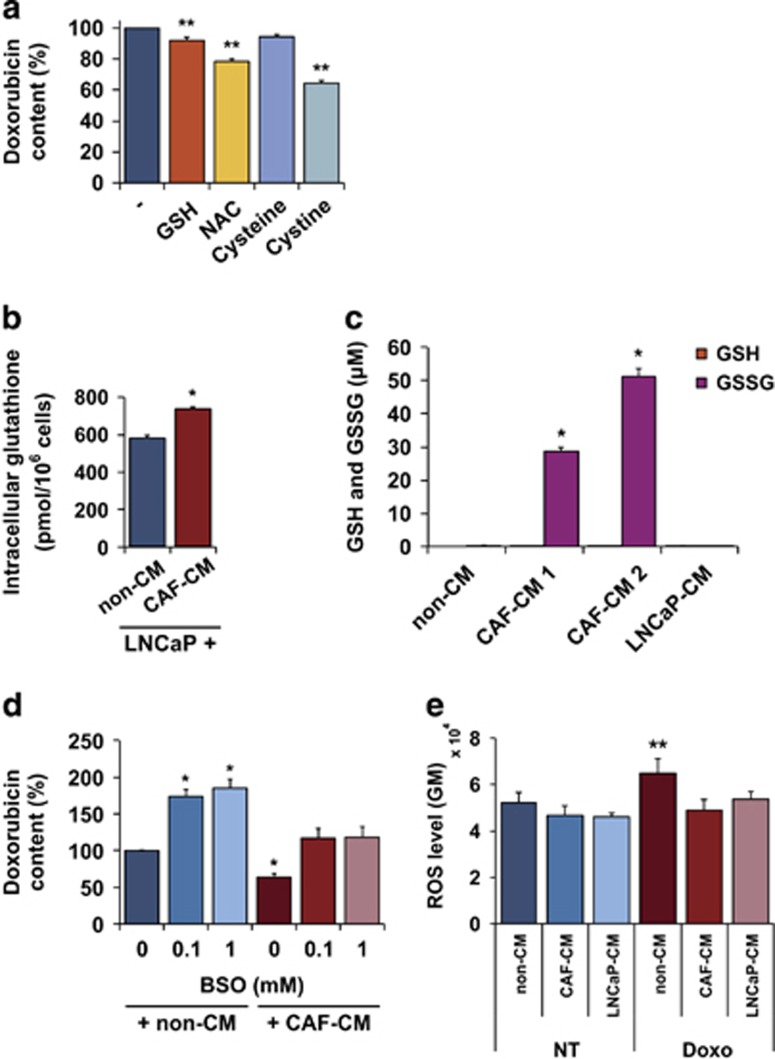
Role of glutathione and its precursors for doxorubicin accumulation in LNCaP cells. (**a**) Reduction in doxorubicin content in LNCaP cells after exposure to 5 mM reduced glutathione (GSH) or *N*-acetyl-l-cysteine (NAC) or 1 mM cysteine or cystine daily for 3 days, and 1 *μ*M doxorubicin for 8 h, in relation to doxorubicin-treated only control (mean and S.E.M.; *N*=5; **P<0.01). (**b**) Total intracellular glutathione levels (GSH+GSSG) in LNCaP cells cultured in fresh (non-CM) or CAF-conditioned medium (CAF-CM), as determined by the glutathione assay described in the Materials and Methods' section (mean and S.E.M.; *N*=6; *P<0.05). (**c**) GSH and GSSG concentrations in different media assessed by HPLC (mean and S.E.M.; *N*=2; *P<0.05). Two different pools of CAF-conditioned medium were tested. (**d**) Effect of l-Buthionine Sulphoximine (BSO) on doxorubicin accumulation in LNCaP cells in the presence or absence of CAF-CM. BSO was added to the culture medium from day 1. After 3 days of culture with conditioned medium in the presence or absence of BSO as indicated, the cells were exposed to 1 *μ*M doxorubicin for 8 h (mean and S.E.M.; *N*=3; *P<0.05). (**e**) ROS levels in LNCaP cells after 3 h treatment with 1 *μ*M doxorubicin as assessed by CellROX and expressed as geometric mean fluorescence intensity (mean and S.E.M.; *N*=3; **P<0.01). The cells were cultured in fresh (non-CM), CAF-conditioned medium (CAF-CM) or LNCaP-conditioned medium (LNCaP-CM)

Small molecules such as cAMP and PGE_2_ have been shown to attenuate DNA damage-induced accumulation of p53 in BCP-ALL cells and inhibit apoptosis.^23, 24^ However, we did not find any inhibitory effect of the cAMP analog 8-CPT-cAMP, the adenylyl cyclase activator forskolin, or PGE_2_ on doxorubicin accumulation in LNCaP cells (data not shown). Other studies have indicated that stromal cells can secrete thiol-containing compounds that promote chemoresistance and cancer cell survival.^25, 26^ We therefore tested the effect of GSH, NAC, cysteine and cystine at different concentrations on doxorubicin accumulation in LNCaP cells. Incubation with NAC and cystine, both precursors of glutathione, led to a marked decrease in the doxorubicin content in a dose-dependent manner (Supplementary Table 1). As shown in Figure 5a, intracellular doxorubicin accumulation was reduced by 20% at the highest concentration of NAC (5 mM) and almost 40% with cystine (1 mM) as compared to control cells. Reduced glutathione (GSH) and the GSH precursor cysteine caused a more modest reduction of doxorubicin accumulation (around 10% and 5%, respectively) at the highest concentrations (Figure 5a).

Next, we tested whether CAF-conditioned medium had any effect on glutathione levels in LNCaP cells. LNCaP cells grown in CAF-conditioned medium showed a 30% increase in intracellular glutathione levels as compared with LNCaP cells cultured with non-conditioned medium (Figure 5b). As glutathione reductase was used in the GSH assay, both reduced (GSH) and oxidized (GSSG) forms were assessed. However, levels of GSSG were as low as 4 pmol per 10^6^ cells (Supplementary Figure 7A), compared with 600–800 pmol per 10^6^ cells total glutathione.

We also examined the thiol contents of CAF-conditioned medium by HPLC. As shown in Figure 5c and Supplementary Figure 7B, this analysis revealed elevated levels of oxidized glutathione (GSSG) in CAF-conditioned medium (30–50 *μ*M), as compared with the almost undetectable levels in non-conditioned RPMI 1640 or LNCaP-conditioned medium. Cysteine was not detected in any of the media, and cystine levels were similar in CAF-conditioned medium and LNCaP- or non-conditioned medium (Supplementary Figures 7B and C).

To study a possible role of GSH further, we inhibited glutathione synthesis in LNCaP cells with l-buthionine sulphoximine (BSO), a glutamate-cysteine ligase (GCL) inhibitor. This resulted in enhanced doxorubicin content both in the presence and absence of CAF-conditioned medium (Figure 5d). The intracellular doxorubicin level in LNCaP cells treated with CAF-conditioned medium together with BSO was comparable to that of cells treated with non-conditioned control medium without BSO (*P*-value=0.3). However, the doxorubicin content of cells in the presence of BSO treatment and CAF-conditioned medium was not as high as in cells exposed to non-conditioned medium and BSO, suggesting that interference with GCL alone is not sufficient to abrogate the CAF-mediated protective effect. Presumably, other factors are also important for CAF-mediated inhibition of doxorubicin accumulation. Taken together, these findings indicate that the CAF-mediated protection against drug-induced cell death is exerted by modulation of GSH levels in the tumor cells.

### CAF-conditioned medium inhibits drug-induced ROS

Both doxorubicin and taxol can generate ROS that leads to increased oxidative stress and cell death.^27, 28^ To examine the effect of CAF-conditioned medium on oxidative stress induced by doxorubicin or taxol, we assessed total ROS levels in LNCaP cells using CellROX deep red fluorescent reagent. Following a 3 h treatment with doxorubicin (Figure 5e) or taxol (Supplementary Figure 8A), ROS levels were significantly elevated in LNCaP cells grown in non-conditioned medium. In contrast, we did not observe any significant induction of ROS in LNCaP cells grown in CAF-conditioned medium after treatment with doxorubicin or taxol. ROS levels in untreated cells were slightly lower in the presence of CAF- or LNCaP-conditioned medium (Figure 5e and Supplementary Figure 8A). LNCaP-conditioned medium also decreased ROS levels upon drug treatment but not to the same extent as CAF-conditioned medium. The known ROS inducer TBHP (200 *μ*M), used as positive control, strongly induced ROS in LNCaP cells after 1 h, and cells that were not stained with CellROX showed very low background staining, confirming that neither doxorubicin nor taxol gave any fluorescence by themselves in the FACS channel used in the assay (Supplementary Figure 8B). Altogether, our data indicate that CAF-conditioned medium protects cancer cells against oxidative stress by modulating cellular ROS levels and scavenging free radicals produced by chemotherapeutic drugs.

## Discussion

**Figure 6:**
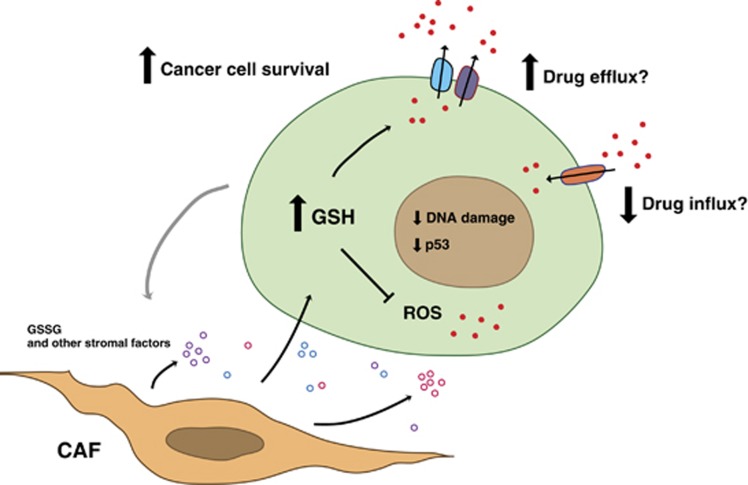
CAFs promote drug resistance and cancer cell survival. Model for interactions between CAFs and cancer cells that lead to enhanced drug resistance. CAFs provide tumor cells with oxidized glutathione as well as other stromal factors. The components are taken up by cancer cells, leading to elevated intracellular GSH levels, inhibition of drug accumulation and inhibition of reactive oxygen species (ROS). CAF-mediated reduction of drug accumulation could possibly be due to decreased influx and/or increased efflux. As a result, DNA damage and p53 induction are attenuated, and cancer cell survival is increased

Emerging drug resistance is a major reason for failure of chemotherapy. Although many cancer drugs may show potent antitumor effect at the first cycle of therapy, tumors often gradually develop resistance leading to relapse. To date, most studies have focused on resistance mechanisms related to tumor cell-autonomous signaling pathways. However, there is accumulating evidence that the tumor microenvironment has a critical role in drug resistance. In this study, we have examined the effect of fibroblasts on the response of prostate cancer cells to chemotherapeutic drugs. We found that CAFs from prostate tumors, or conditioned medium derived from these cells, promote chemoresistance by preventing doxorubicin accumulation and ROS induction in the tumor cells. Our data suggest that this is mediated at least in part by CAF-derived glutathione that is taken up by cancer cells. The enhanced intracellular glutathione levels decrease both oxidative stress and accumulation of doxorubicin, attenuate drug-induced DNA damage, p53 induction and cell death, and thus enhance cancer cell survival (Figure 6). These findings are consistent with previously published studies demonstrating a pivotal role of tumor microenvironment and the glutathione pathway in protecting cancer cells against chemical and oxidative stress, along with development of drug resistance.^29, 30, 31^ CAF-derived GSH and cysteine can decrease platinum drug accumulation and contribute to drug resistance in ovarian cancer cells.^25^ Bone marrow stromal cells are able to convert cystine to cysteine, and release it to neighboring leukemia cells, resulting in enhanced glutathione synthesis and thus cancer cell survival.^26^ The reported concentrations of GSH and cysteine in CAF/stromal cell-conditioned medium were in the same range as the concentration of GSSG detected in CAF-CM in our study (30–100 *μ*M).

We have shown that CAFs inhibit accumulation of doxorubicin in the tumor cells and enhance intracellular levels of GSH. This suggests that GSH either inhibits doxorubicin influx or promotes doxorubicin efflux. Doxorubicin and taxol are known to be exported by the multidrug resistance 1 protein (MDR1) and multidrug resistance-associated proteins, such as MRP1 and MRP2.^32^ MDR1 is considered to have a minor role for drug resistance in prostate tumor cells.^33^ On the other hand, MRP family members are expressed in prostate cancer cells,^34^ and we could detect MRP1 protein in LNCaP and 22Rv1 cells by Western blotting (data not shown). Interestingly, GSH has been shown to facilitate drug export mediated by the MRP proteins in tumor cells.^35, 36, 37^ Moreover, GSH can stimulate the ATPase activity of MRP1,^38^ and GSH can also form drug conjugates that are exported.^39, 40^ There is no evidence to date that doxorubicin or taxol can form conjugates with GSH, but MMC is known to conjugate with GSH.^41^ For this reason, CAF could potentially stimulate export of doxorubicin and taxol as unmodified drugs and MMC as a GSH–MMC conjugate.

GSH could also inhibit the effect of chemotherapeutic drugs by a different mechanism. Glutathione is a major antioxidant against oxidative stress, and both doxorubicin and taxol have been shown to induce high levels of ROS and oxidative stress.^27, 28^ These high ROS levels are counteracted by elevated glutathione levels in the tumor cells in the presence of CAFs. Hence, it appears that increased GSH can promote cancer cell survival both by affecting drug influx/efflux and counteracting drug-induced ROS.

Cancer cells, such as LNCaP, express the membrane enzyme *γ*-glutamyl transpeptidase (GGT),^42^ which catalyzes extracellular degradation of GSSG/GSH to its precursor amino acids^43, 44^ that are subsequently transported into the cells, consistent with the idea that GSSG derived from CAFs may act as a source of glutathione precursors and contribute to the enhanced GSH levels in LNCaP cells. In fact, many tumors, including prostate tumors, express high levels of GGT on their cell membranes^45^ and this has been correlated with drug resistance. For instance, increased GGT activity was reported in an ovarian cancer cell line derived from a patient that acquired resistance during treatments with cisplatin, chlorambucil and 5-fluorouracil, compared to another cell line from the same patient before onset of drug resistance.^46^ Furthermore, GGT has an essential role in protecting cells against oxidative challenge. Elevated levels of GGT and increased intracellular glutathione content in rat lung epithelial cells were accompanied by cell adaptation to oxidative stress induced by tert-butylhydroquinone.^47^ Thus, GGT supplies cancer cells with substrates that are necessary for glutathione synthesis to counteract ROS. Although glutamate, glycine and cysteine/cystine are required for GSH synthesis, the cellular cysteine/cystine ratio itself has also been shown to be important in conferring resistance to cell death without altering GSH levels.^48^

The enhanced levels of oxidized glutathione observed in CAFs may be the result of their cross talk with cancer cells. It has been suggested that normal stromal fibroblasts can be instructed by adjacent tumor cells to transform into CAFs by shifting their metabolism towards aerobic glycolysis. In turn, CAFs secrete and provide tumor cells with energy-rich metabolites and chemical building blocks such as amino acids,^49^ and are fixed in this state without being able to switch back to the normal phenotype. In addition, enhanced oxidation in tumor cells may result in export of ROS, that is, hydrogen peroxide,^50^ and induction of oxidative stress in the stromal cells.^51^ Consistent with this notion, we have observed excretion of hydrogen peroxide by LNCaP cells (data not shown). Indeed, induction of oxidative stress in tumor stroma was shown to promote increased production of glutathione species, including amino acid metabolites associated with the glutathione pathway, such as cysteine, glycine and glutamate.^52^

Our data show that both CAFs and NFs can modulate p53 levels and survival of LNCaP cells. Notably, HDFs and hTERT-immortalized fibroblasts that have never been exposed to a tumor microenvironment did not affect doxorubicin accumulation and tumor cell survival. CAFs were in general more potent than NFs. The indication of partial CAF-like properties of the NFs are consistent with previous studies, which have demonstrated an activated, and clinically informative, phenotype of fibroblasts in histologically normal areas of cancer-affected prostates.^53, 54^

In summary, our results reveal that activated fibroblasts of the tumor stroma promote drug resistance and support survival of prostate carcinoma cells by elevating the intracellular level of GSH, and thus attenuating both drug accumulation and ROS induction. Therapeutic targeting of CAFs is an appealing strategy, given the fact that CAFs are more genetically stable than tumor cells and probably less likely to acquire therapy resistance. Inhibitors that cause thiol/redox imbalance would decrease GSH in cancer cells, and consequently block the protection against chemotherapy. Novel cancer therapy targeting CAFs and the intercellular metabolic pathway in combination with conventional therapies may allow more efficient treatment of drug-resistant tumors.

## Materials and methods

### Cell culture

Primary human prostate fibroblast cultures were established as described previously.^55^ In brief, fresh prostate tissue pieces of about 1 mm^3^ were harvested from grossly malignant and benign areas of the cut surfaces of radical prostatectomy specimens. For morphological control, cell smears from these areas were stained by Giemsa and whole-mount histological sections were reviewed. The 1 mm^3^ tissue pieces were put into six-well tissue culture plates and fixed in the well under a cover slide. Then 1.5 ml Bfs medium (DMEM (Hyclone, Logan, UT, USA) supplemented with 5% FBS (Hyclone), 5% Nu Serum (BD Biosciences, Stockholm, Sweden), 5 *μ*g/ml Insulin, 0.5 *μ*g/ml Testosterone, 2 mM l-glutamine and 1 × penicillin/ streptomycin (all Sigma-Aldrich, Stockholm, Sweden) was added to each well and the tissue pieces were incubated at 37 °C with 5% CO_2_. Fibroblast-like cells started to migrate out from the tissue between 5 and 15 days and were passaged when confluent. The fibroblast nature of the tissue-derived cell cultures was verified by their fibroblast-characteristic morphology and the expression of fibroblast markers such as PDGFR-b, *α*-SMA but not, for example, E-cadherin. Cultures at passages 5–13 were used in this study and grown in the same medium. Fibroblasts from malignant areas were termed cancer-associated fibroblasts (CAFs), and those from benign areas were termed normal fibroblasts (NFs).

LNCaP and 22Rv1 prostate carcinoma cells were purchased from the American Type Culture Collection (ATCC, Manassas, VA, USA) and grown in RPMI 1640 medium (Hyclone) supplemented with 10% fetal bovine serum (Hyclone), 2 mM l-glutamine (Hyclone) and 2.5 *μ*g/ml plasmocin (InvivoGen, Toulouse, France). BJ hTERT-immortalized fibroblasts were cultured in DMEM high-modified medium (Hyclone) supplemented with 10% fetal bovine serum, 4 mM l-glutamine and 2.5 *μ*g/ml plasmocin, and human dermal fibroblasts (HDFs) in DMEM low-glucose medium (Hyclone) supplemented with 10% fetal bovine serum and 2.5 *μ*g/ml plasmocin. All the cells were maintained in 5% CO_2_ and 100% humidity at 37 °C, and were tested regularly and negative for mycoplasma.

For transwell co-culture assays, LNCaP cells were plated in six-well plates and allowed to adhere overnight in RPMI 1640 medium. Fibroblasts or LNCaP cells were plated in cell culture inserts using the same medium and placed on top into the same six-well plates after overnight adhering. The cells were incubated together for 2.5 days before drug treatments. At the time of treatment, the tumor stroma ratio was 2:1.

To generate conditioned media, fibroblasts or LNCaP cells were plated in six-well plates at different cell densities. After allowing the cells to adhere overnight, the media were replaced with RPMI 1640 medium in a final volume of 2 ml/well, and after 3 days, all cells had reached equal density of 4 × 10^5^ cells/well. The conditioned media were then collected, sterile filtered through 0.2 *μ*m membrane and aliquots were stored at −80 °C. LNCaP or 22Rv1 cells were cultivated in different conditioned media or non-conditioned medium (fresh RPMI 1640) for 2.5 days before drug treatments. For both co-culture and conditioned medium assays, CAFs and NFs were composed of pools of fibroblasts from three to four patients.

### Antibodies and reagents

The cells were treated either with 1 *μ*M doxorubicin, 20 nM Taxol (Paclitaxel), 6 *μ*g/ml MMC (all Sigma-Aldrich), or with different concentrations of *N*-acetyl-cysteine, l-cysteine, cystine, reduced glutathione and buthionine sulfoximine (Sigma-Aldrich). The primary antibodies used were anti-p53 DO-1 mouse monoclonal (Santa Cruz Biotechnology, Santa Cruz, CA, USA, Cat. # sc-126), anti-GAPDH FL-335 rabbit monoclonal (Santa Cruz, Cat. # sc-25778) and anti-phospho-Histone H2AX Ser139 (Merck Millipore, Stockholm, Sweden, Cat. # 05-636 clone JBW301).

### Sub-G1 assay by FACS

For sub-G1 analysis, 2 × 10^5^ of LNCaP cells were plated in six-well plates, adhered overnight and then placed in co-culture or incubated with conditioned media for 2.5 days. All the cells were collected after 24, 48 and 72 h of drug treatments, washed with PBS and pelleted. The cell pellets were re-suspended in 0.75 ml cold PBS, and then 1 ml of 99% cold ethanol was added dropwise while vortexing. After overnight fixation at 4 °C, the cells were centrifuged, re-suspended, stained with 0.05 mg/ml propidium iodide (PI) (Sigma-Aldrich) and treated with 0.25 mg/ml RNAse A (Sigma-Aldrich) at 37 °C for 30 min. Additional PBS was later added to the samples and the cells were analyzed on a BD FACSCalibur System. Approximately 10 000 cells were used in the analysis for each experiment.

### Immunofluorescence

A total 1 × 10^4^ fibroblasts were seeded on eight-well chamber slides per well. After overnight adhering, 2 × 10^4^ LNCaP-eGFP cells were then seeded onto the fibroblasts, for a direct-contact co-culture. After 2.5 days of co-culture and additional 9 h of drug treatment, the cells were washed with PBS, fixed with 4% formaldehyde for 10 min and permeabilized with 0.2% Triton for 2 min. The fixed cells were later incubated with the anti-p53 primary antibody (1:300) at 4 °C overnight. The next day, cells were washed with PBS and then incubated with anti-mouse Alexa Fluor 594 conjugated secondary antibody (1:1000; Life Technologies, Stockholm, Sweden) an additional hour at room temperature. The cells were finally washed with PBS and mounted using Vectashield Hard Set Mounting Medium containing DAPI (Vector Laboratories, Burlingame, CA, USA). The images were captured with LNCaP cells in focus on a Zeiss AxioPlan 2 microscope connected to an AxioCam HRm microscope camera. More than 90 cells were analyzed with Adobe Photoshop for each experiment.

For Operetta high content imaging system analysis, the cells were seeded at 2 × 10^4^ cells/well on poly-l-lysine (Sigma-Aldrich) pre-coated 96-well plates. The cells were prepared in a similar manner as described above and were incubated with the anti-*γ*H2AX primary antibody (1:500) and anti-mouse Alexa Fluor 488 conjugated secondary antibody (1:1000; Life Technologies). DNA was then stained with DAPI (Sigma-Aldrich). Images on more than 3000 cells acquired on the Operetta system were analyzed with Columbus software (Perkin-Elmer, Waltham, MA, USA) and each sample was analyzed in triplicate.

### Western blotting

LNCaP cells were seeded at 4 × 10^5^ cells/well and 22Rv1 at 1.5 × 10^5^ cells/well in six-well plates, adhered overnight and then placed in co-culture or incubated with conditioned media for 2.5 days. After drug treatments, whole-cell lysates were prepared with lysis buffer; containing 100 mM Tris pH 7.4, 150 mM NaCl, 1% NP 40, 1% protease inhibitor cocktail (Sigma-Aldrich), and the protein concentrations were determined by Bradford assay. 25 *μ*g of proteins were heated at 95 °C for 10 min and separated by SDS-PAGE using 10% Bis-Tris polyacrylamide gels (Life Technologies) in 1 × MOPS buffer (Life Technologies). The proteins were thereafter transferred to nitrocellulose membranes using iBlot (Life Technologies). After blocking, the membranes were immunoblotted with either anti-p53 or anti-GAPDH primary antibody (1:1000) at 4 °C overnight and probed with anti-mouse or anti-rabbit HRP-conjugated secondary antibody (1:10 000) the next day for 1 h at room temperature. Finally, the blots were detected with Super Signal West Femto Maximum Sensitivity Substrate (Thermo Scientific, Stockholm, Sweden) and a CCD camera (Fujifilm, Stockholm, Sweden). GAPDH served as loading control. The samples that were compared were loaded on the same gel and transferred onto the same membrane.

### Cellular drug content assays

LNCaP cells were seeded at 4 × 10^5^ cells/well and 22Rv1 at 1.5 × 10^5^ cells/well in six-well plates, adhered overnight and then incubated with conditioned media for 2.5 days before doxorubicin treatment. Doxorubicin-treated cells were collected, washed twice with ice-cold PBS, and re-suspended in PBS containing Ca^2+^ and Mg^2+^. The intracellular doxorubicin accumulation was assessed using a NovoCyte flow cytometer, except in Figure 4a, where BD LSRII flow cytometer was used. The cells were analyzed with excitation at 488 nm and emission integrated at 530 nm, or 615 nm for BD LSRII. A total 10^4^ cells were included in the analysis for each experiment.

For taxol content, LNCaP cells were seeded at 5 × 10^5^ cells/well in six-well plates, adhered overnight and then incubated with conditioned media for 2.5 days before treatment with taxol. The cells were then exposed to 50 nM taxol for 30 min. Thereafter, the drug was removed, and the cells were washed twice and cultured further in drug-free media. After 4 and 6 h of drug removal, the cells were washed and collected. After re-suspending the cells in hypotonic lysis buffer (10 mM Tris-HCL pH 7.5), the cells were exposed to repeated freeze/thaw cycles and centrifuged at 14 000 r.p.m. for 10 min. Supernatant and pellet were collected separately, extracted twice with ethyl acetate and concentrated under vacuum in SpeedVac (Thermo Scientific, Stockholm, Sweden). The samples were then dissolved in 20 *μ*l of acetonitrile and stored at −20 °C before performing the analysis on a Beckman HPLC instrument operated with System Gold coupled to diode array UV–VIS detector module 168 (a kind gift from Associate Professor Dan Segerbäck). Chromatography was carried out on a Luna C18(2) column (Phenomenex, Copenhagen, Denmark; 4.6 × 250 mm, 5 *μ*m particle size) with a pre-column filter. The separation was performed using a gradient elution with water mixed with acetonitrile. The initial elution was isocratic with 50% acetonitrile for 5 min, followed by a linear gradient for 15 min up to 100% acetonitrile. Then the column was washed with acetonitrile for 10 min followed by a linear gradient back to the initial mixture with 50% acetonitrile. The flow rate was 1 ml/min. The detector was set to 225 nm absorbance measurements. For the calculation of product values, HPLC peak areas were integrated with OriginPro 8.5 software (Northampton, MA, USA). Serial dilutions of taxol standard were used for estimation of its concentration in the samples.

### Fractionation of conditioned medium

CAF-, LNCaP- and non-conditioned medium were fractionated using Amicon Ultra-15 Centrifugal Filter Device with a 10 kDa or 3 kDa Nominal Molecular Weight Limit membrane cut-off (Millipore). For three loaded samples, three additional columns of non-conditioned medium were fractionated and served to mix with different fractions (complemental liquid). First, 5 ml of media were loaded onto each filter device and centrifuged at 3 000 g at 4 °C for 90 min. Then, the solute (>10 K or >3 K fraction) was re-suspended in the eluate of complemental liquid, which was the same volume as the loaded sample. The eluate (<10 K or <3 K fraction) was collected and mixed with the solute of complemental liquid. All fractions were used immediately after preparation. To exclude the loss of components during the fractionation procedure, the solute and eluate of CAF-CM were re-pooled together and tested.

### Detection of intracellular glutathione

The content of total glutathione in cell lysates were determined using an enzymatic recycling method. The assay based on the reaction of GSH with 5,5′-dithio-bis-2-nitrobenzoic acid (DTNB) in the presence of glutathione reductase, was performed according to the protocol^56^ and measured with a TECAN plate reader at 412 nm. The concentrations of glutathione were then calculated from slopes of the absorbance changes in reference to the standard curve. For GSSG measurement, 2-vinylpyridine (Sigma-Aldrich) was added before the enzymatic reactions to remove reduced glutathione. A total 2 × 10^5^ cells were used in each experiment for detection of total glutathione and 10^6^ cells for GSSG measurement.

### Detection of glutathione in cell culture medium

A total 20 *μ*l of CAF-, LNCaP- or non-conditioned medium was mixed with 50 *μ*l acetone and incubated at −20 °C overnight. The samples were thereafter centrifuged at 14 000 r.p.m. at 4 °C for 10 min, and supernatant was collected, re-centrifuged again and transferred to the new tube. Next, the samples were concentrated in SpeedVac (Savant) under vacuum for 40 min, and the concentrated samples were re-constituted to the original volume by 10 *μ*l of Millipore water. Half of the samples were also incubated in 4 *μ*l of 10 mM TCEP (Sigma-Aldrich) at room temperature for 30 min, to reduce oxidized thiols. After derivatization with 10 *μ*l of 5 mM 7-diethylamino-3-(4-maleimidophenyl)-4-methylcoumarin (Sigma-Aldrich) at room temperature for 10 min, the maleimide ring was cleaved by addition of 30 *μ*l of 50 mM ammonium formate (Sigma-Aldrich) pH 4.2 at room temperature for another 30 min. Each sample was thereafter incubated with 10 *μ*l of acetonitrile and stored at −20 °C before analysis was performed on a Beckman HPLC instrument operated with System Gold coupled to diode array UV–VIS detector module 168. Chromatography was carried out on a Luna C18(2) column (Phenomenex; 4.6 × 250 mm, 5 *μ*m particle size) with a pre-column filter. The separation was performed using a gradient elution with water mixed with acetonitrile. The initial elution was isocratic with 30% acetonitrile for 2 min, followed by a linear gradient for 15 min up to 100% acetonitrile. The column was washed with acetonitrile for 10 min followed by a linear gradient back to the initial mixture of 30% acetonitrile with the flow rate 1 ml/min. The product values were calculated on HPLC peak areas and integrated with OriginPro 8.5 software (Northampton, MA, USA).

### Intracellular ROS measurement

LNCaP cells were seeded at 6 × 10^4^ cells/well in 24-well plates, adhered overnight and then incubated with conditioned media for 2.5 days before treatment with doxorubicin or taxol. Following 3 h of drug treatments, 5 *μ*M of the cell-permeable, ROS-sensitive dye CellROX Deep Red (Invitrogen, Stockholm, Sweden) was added to each sample and incubated at 37 °C for 30 min. The cells were collected, washed and ROS was quantified on a NovoCyte flow cytometer, with an excitation max of 640 nm and emission max of 665 nm. A total 10^4^ cells were included in the analysis for each experiment. The cells treated for 1 h with 200 *μ*M of the ROS inducer TBHP were used as a positive control.

### Statistical analysis

All experiments were performed independently at least three times and the data were shown as mean and S.E.M. *N* is the number of times each experiment was repeated. Statistical analysis was performed using two-tailed, paired *t*-test by comparing all the samples to control sample that is non-CM or monoculture. All *P*-values <0.05 were considered significant.
