# Supplementary material for: Human cancer-associated fibroblasts enhance glutathione levels and antagonize drug-induced prostate cancer cell death
Source: Cell Death Dis. 2017 Jun 1;8(6):e2848–. doi: 10.1038/cddis.2017.225 (PMC5520886; doi:10.1038/cddis.2017.225)
Supplement: Supplementary Figures and Table [file cddis2017225x1.pdf]

Supplementary Figures

Figure S1

A

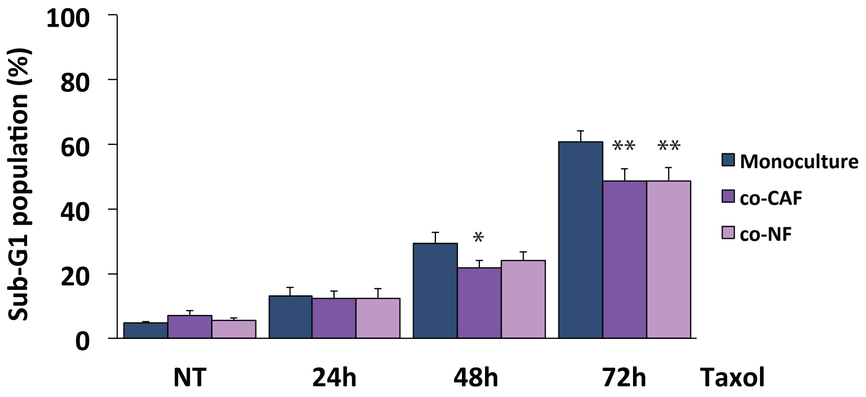

B

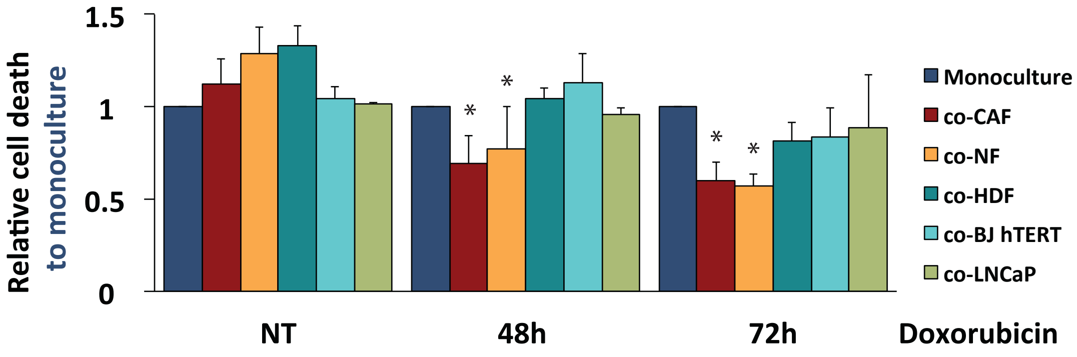

Figure S2

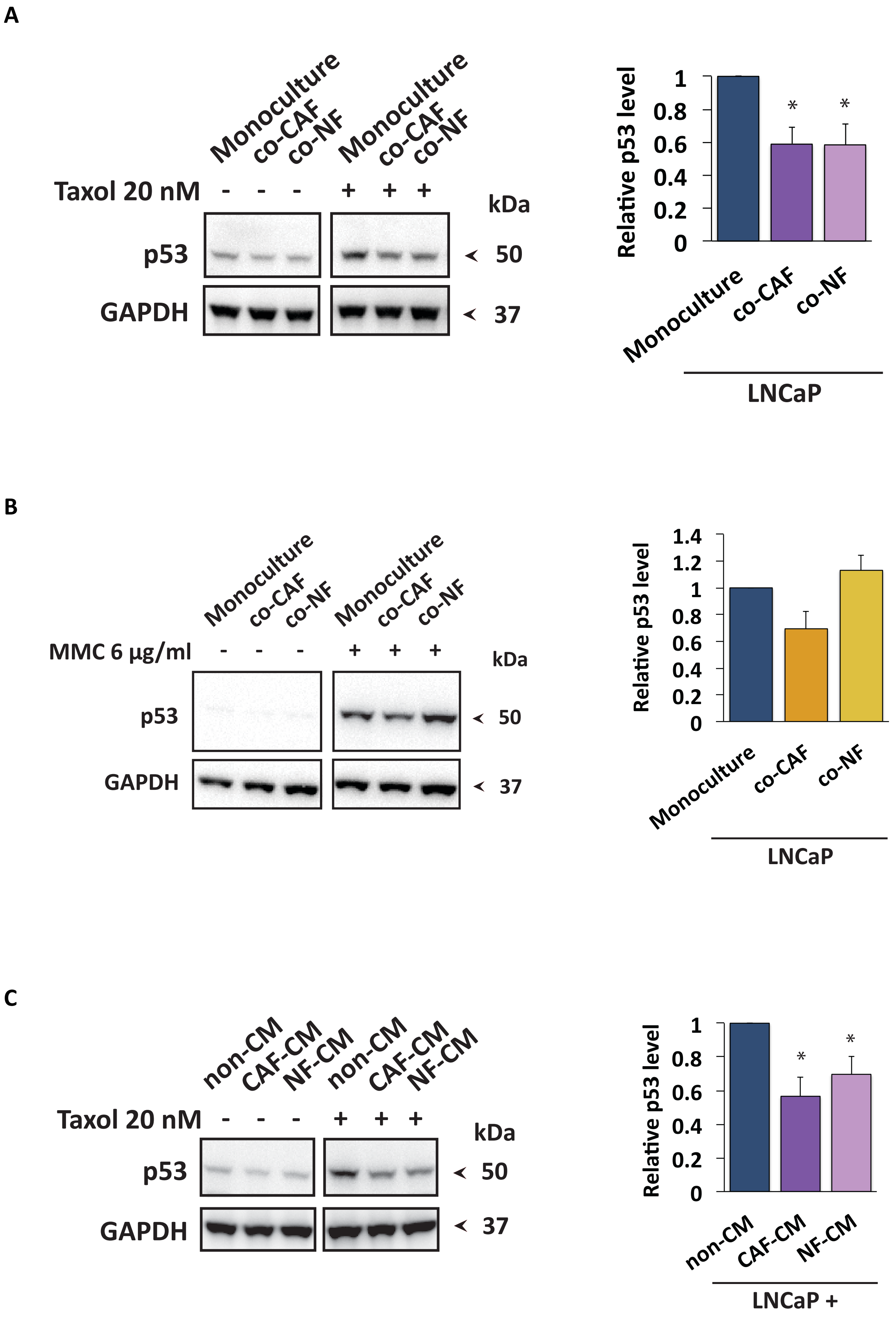
A
Monoculture
co-CAF
co-NF
Monoculture
co-CAF
co-NF
Taxol 20 nM - - - + + +
kDa
p53
50
GAPDH
37
Relative p53 level
1
0.8
0.6
0.4
0.2
0
*
*
Monoculture
co-CAF
co-NF
LNCaP
B
Monoculture
co-CAF
co-NF
Monoculture
co-CAF
co-NF
MMC 6 µg/ml - - - + + +
kDa
p53
50
GAPDH
37
Relative p53 level
1.4
1.2
1
0.8
0.6
0.4
0.2
0
Monoculture
co-CAF
co-NF
LNCaP
C
non-CM
CAF-CM
NF-CM
non-CM
CAF-CM
NF-CM
Taxol 20 nM - - - + + +
kDa
p53
50
GAPDH
37
Relative p53 level
1
0.8
0.6
0.4
0.2
0
*
*
non-CM
CAF-CM
NF-CM
LNCaP +

Figure S3

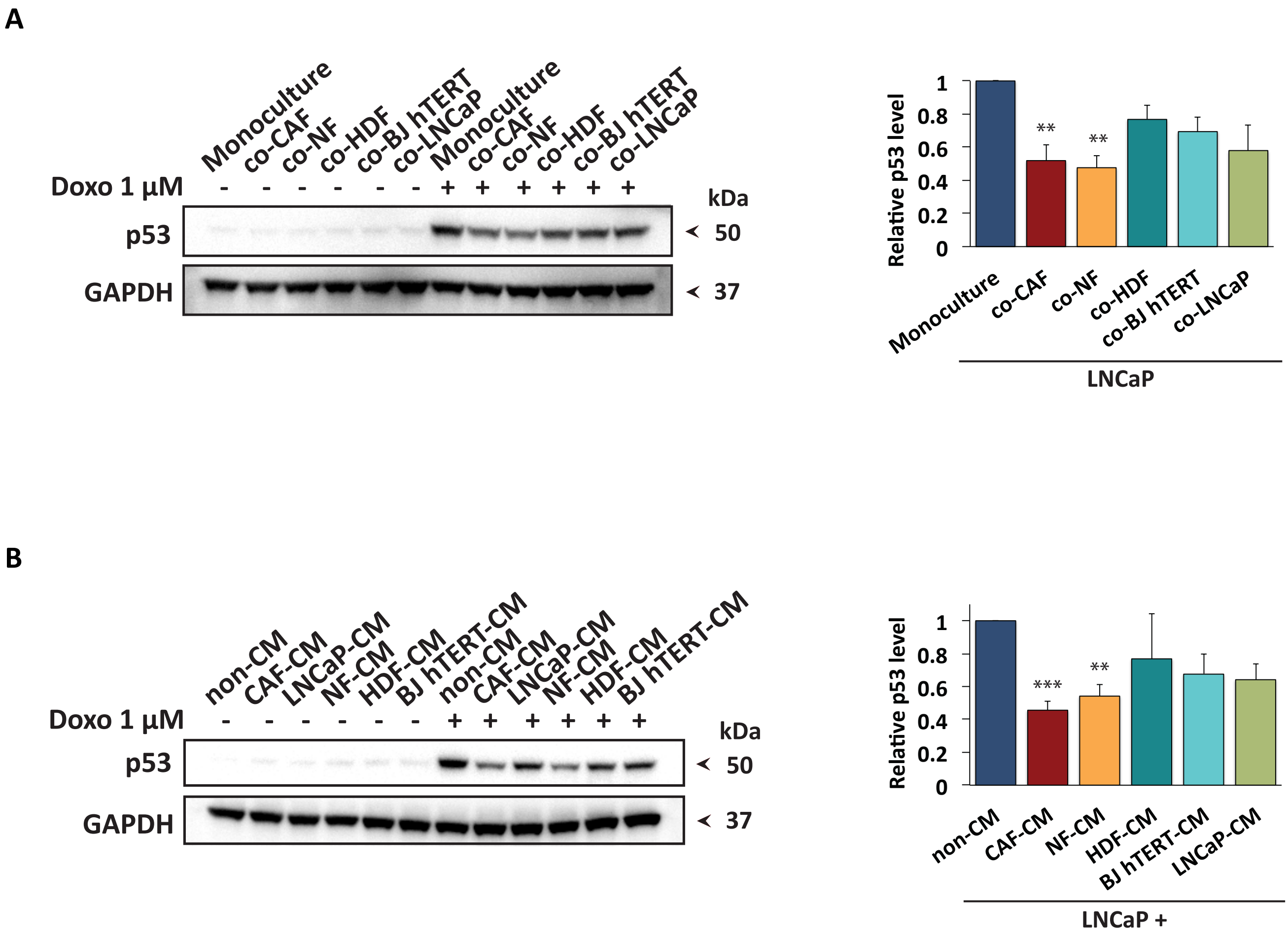
A
Monoculture
co-CAF
co-NF
co-HDF
co-BJ hTERT
co-LNCaP
Monoculture
co-CAF
co-NF
co-HDF
co-BJ hTERT
co-LNCaP
Doxo 1 µM
- - - - - - + + + + + +
kDa
p53
50
GAPDH
37
Relative p53 level
1
0.8
0.6
0.4
0.2
0
**
**
Monoculture
co-CAF
co-NF
co-HDF
co-BJ hTERT
co-LNCaP
LNCaP
B
non-CM
CAF-CM
LNCaP-CM
NF-CM
HDF-CM
BJ hTERT-CM
non-CM
CAF-CM
LNCaP-CM
NF-CM
HDF-CM
BJ hTERT-CM
Doxo 1 µM
- - - - - - + + + + + +
kDa
p53
50
GAPDH
37
Relative p53 level
1
0.8
0.6
0.4
0.2
0
***
**
non-CM
CAF-CM
NF-CM
HDF-CM
BJ hTERT-CM
LNCaP-CM
LNCaP +

Figure S4

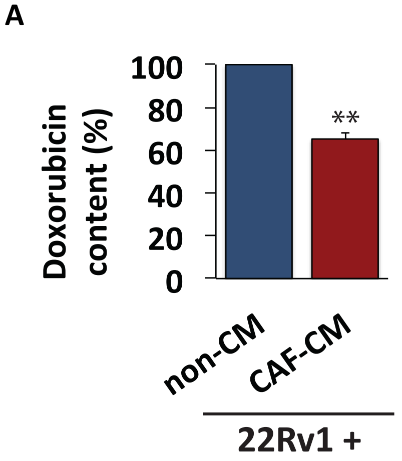
A
Doxorubicin content (%)
100
80
60
40
20
0
**
non-CM
CAF-CM
22Rv1 +

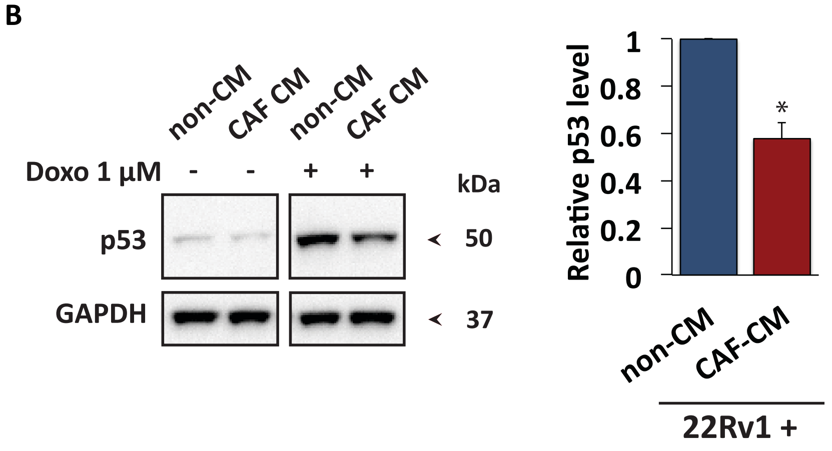
B
non-CM
CAF CM
non-CM
CAF CM
Doxo 1 μM - - + +
kDa
p53
50
GAPDH
37
Relative p53 level
1
0.8
0.6
0.4
0.2
0
*
non-CM
CAF-CM
22Rv1 +

Figure S5

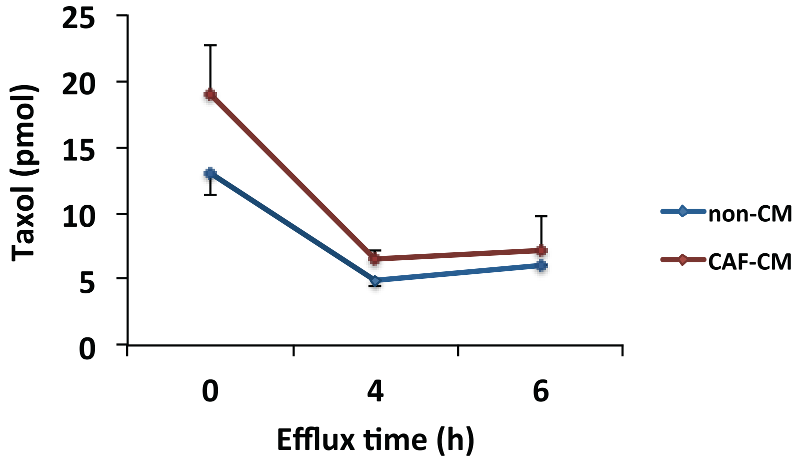

Figure S6

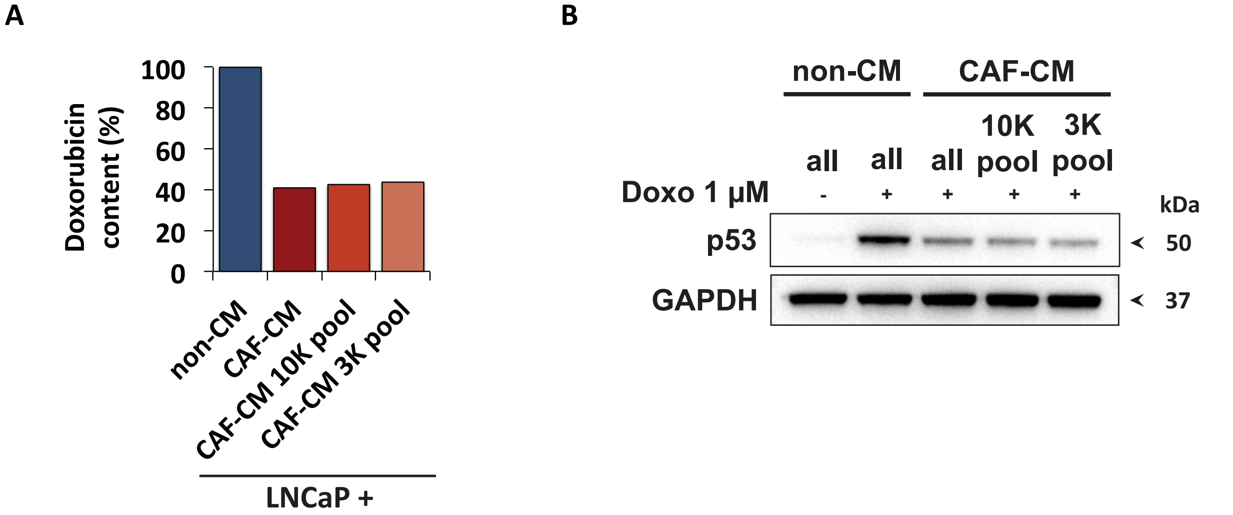
A
Doxorubicin content (%)
100
80
60
40
20
0
non-CM
CAF-CM
CAF-CM 10K pool
CAF-CM 3K pool
LNCaP +
B
non-CM
CAF-CM
all
all
all
10K pool
3K pool
Doxo 1 μM
-
+
+
+
+
kDa
p53
50
GAPDH
37

Figure S7

A

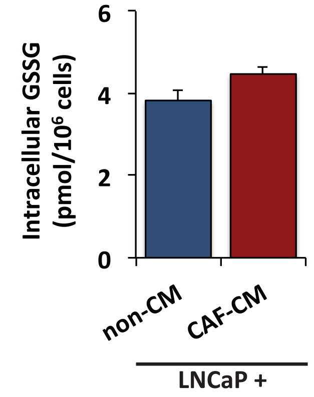

C

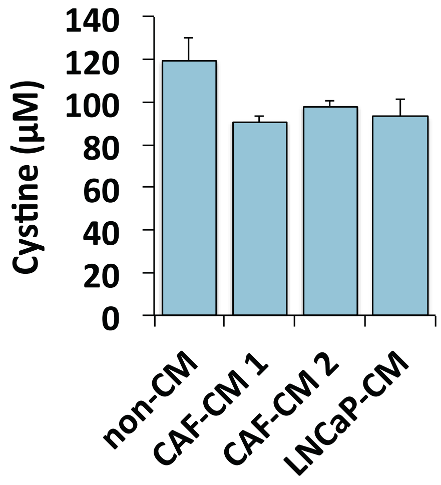

B

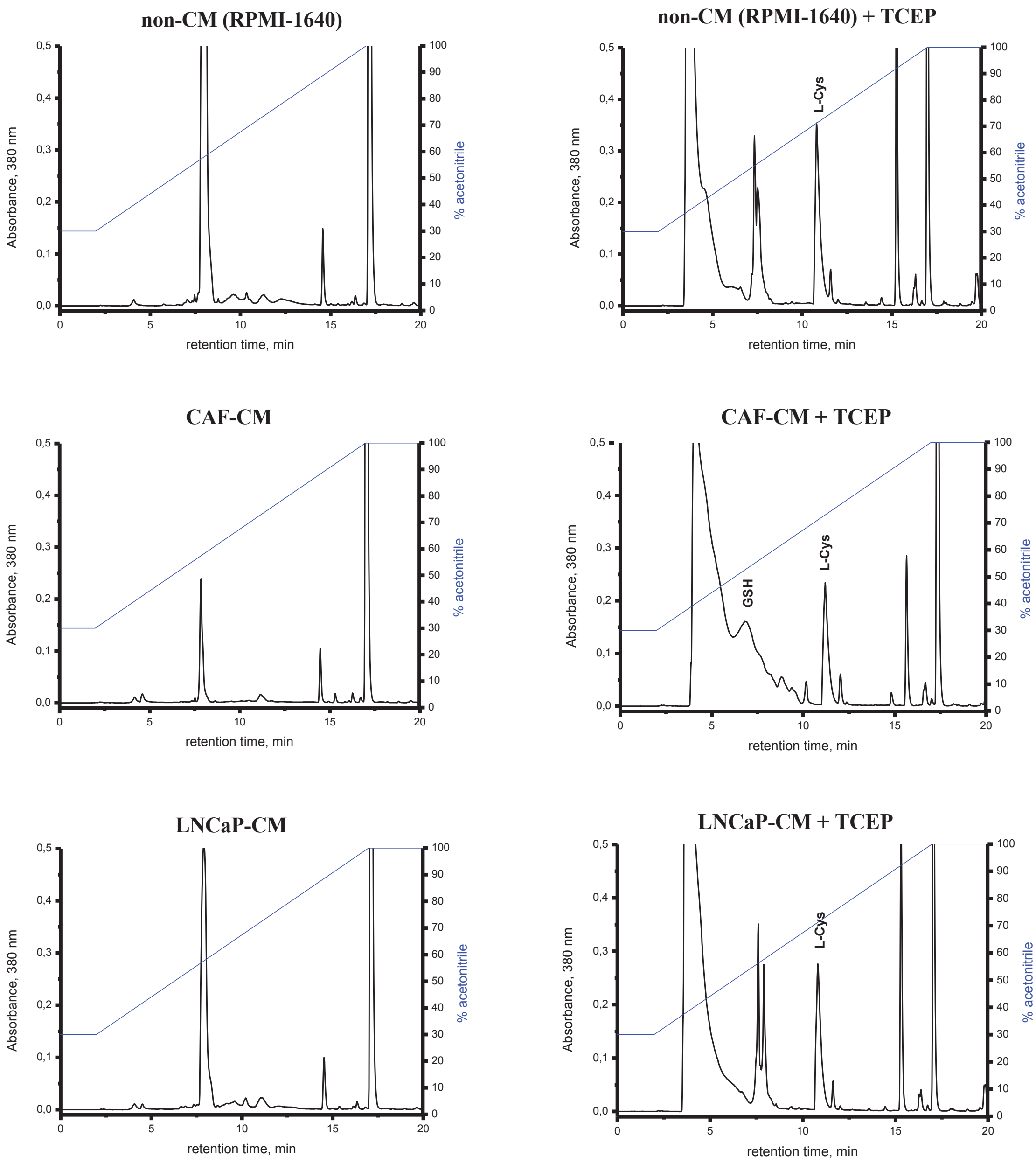

Figure S8

A

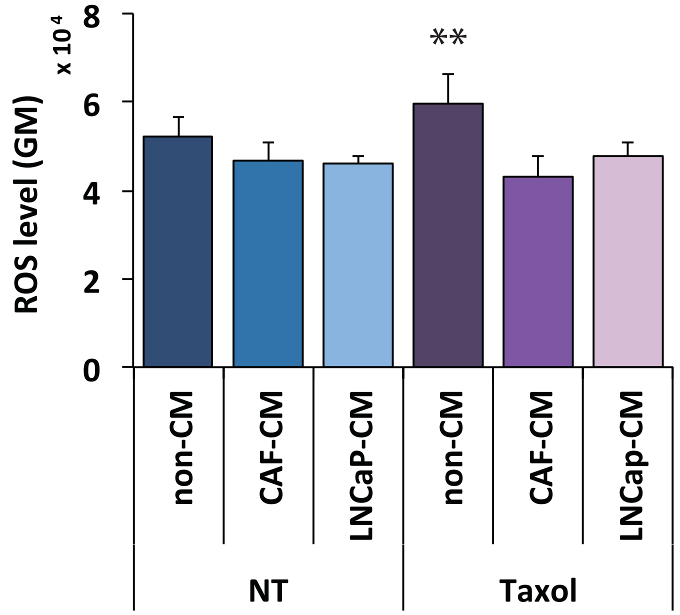
x 10^4
ROS level (GM)
8
6
4
2
0
**
non-CM
CAF-CM
LNCaP-CM
non-CM
CAF-CM
LNCap-CM
NT
Taxol

B

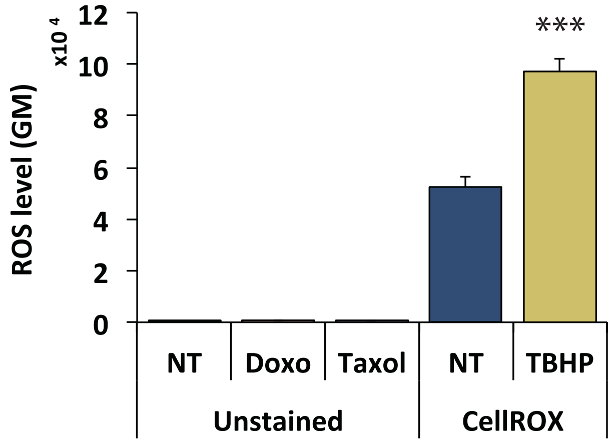
x10^4
ROS level (GM)
12
10
8
6
4
2
0
***
NT
Doxo
Taxol
NT
TBHP
Unstained
CellROX

Supplementary Table

Table S1

	Concentration (mM)	% reduction in doxo content
-	0	0
GSH	0.1	-0.2
	1	3.4
	5	7.9
NAC	0.5	3.5
	5	21.5
Cysteine	0.05	0.7
	0.2	4.4
	1	5.6
Cystine	0.05	1.8
	0.2	5.5
	1	35.6
