# Supplementary material for: Human cancer-associated fibroblasts enhance glutathione levels and antagonize drug-induced prostate cancer cell death
Source: Cell Death Dis. 2017 Jun 1;8(6):e2848–. doi: 10.1038/cddis.2017.225 (PMC5520886; doi:10.1038/cddis.2017.225)
Supplement: Supplementary Figures and Table Legends [file cddis2017225x2.docx]

**Supplementary Information**

**Supplementary Figure Legends**

**Figure S1. CAFs increase LNCaP cell survival after treatment with taxol or doxorubicin.** **A,** Sub-G1 population of LNCaP cells in co-culture with fibroblasts (CAF or NF) using transwell system, as assessed by FACS-PI after 24, 48 and 72 hours of 20 nM taxol treatment (mean and s.e.m.; N=7; *p < 0.05, **p < 0.01). **B,** Cell death of LNCaP co-cultivated in transwell system with various fibroblasts (CAF, NF, HDF or BJ hTERT) or LNCaP themselves in relation to cell death in monoculture conditions of each time point. Sub-G1 population was determined by FACS-PI after 48 and 72 hours treatment with 1 μM doxorubicin (mean and s.e.m.; N=4; *p < 0.05).

**Figure S2. CAFs reduce p53 accumulation induced by taxol or mitomycin C in LNCaP cells.** **A,** Left, a representative immunoblot of p53 induction after 9 hours of 20 nM taxol or **B,** 6 μg/ml MMC treatment in LNCaP cells co-cultured with fibroblasts (CAF or NF) using transwell system, and **C,** cultured with fibroblast- (CAF or NF) conditioned media (CM) in the presence of taxol. Right, quantifications of p53 level after the drug treatments in relation to p53 level of cells cultured alone or in non-conditioned medium together with drug treatments (mean and s.e.m.; N=2 or 5; *p < 0.05).

**Figure S3. CAFs attenuate p53 accumulation induced by doxorubicin. A,** Left, p53 induction after 8 hours of 1 μM doxorubicin treatment in LNCaP cells co-cultured with various fibroblasts (CAF, NF, HDF or BJ hTERT) or LNCaP themselves using transwell system and **B,** in culture with fibroblast- (CAF, NF, HDF or BJ hTERT) or LNCaP-conditioned medium (CM), as indicated by a representative immunoblot. Right, quantifications of relative p53 level after the drug treatments in relation to p53 level of cells cultured alone or in non-conditioned medium together with doxorubicin (mean and s.e.m.; N=3; **p < 0.01, ***p < 0.0001).

**Figure S4. CAF-CM decreases cellular doxorubicin accumulation and p53 response in 22Rv1 cells.** **A,** Doxorubicin content as assessed by FACS, in 22Rv1 cells cultured with CAF-conditioned media (CM) and exposed to 8 hours of 1 μM doxorubicin (mean and s.e.m.; N=4; **p < 0.01). **B,** Left, a representative immunoblot of p53 induction after 8 hours of 1 μM doxorubicin treatment in 22Rv1 cells in culture with CAF-conditioned medium (CM). Right, quantification of relative p53 level after the drug treatment in relation to p53 level of cells cultured in non-conditioned medium and doxorubicin (mean and s.e.m.; N=3; *p < 0.05).

**Figure S5. CAFs do not significantly affect taxol accumulation.** Taxol content in LNCaP cells exposed to CAF- or non-conditioned medium (CM) at 0, 4 or 6 hours after removal of the drug, assessed by HPLC. Cells were exposed to 50 nM taxol for 30 min before the drug is removed. Data presented as mean of a duplicate from one experiment.

**Figure S6. No apparent loss of components from CAF-conditioned medium during fractionation with centrifugal filter devices. A,** Doxorubicin content, as determined by FACS and **B,** p53 induction, as shown by the immunoblot, in LNCaP cells exposed to unfractionated (all) CAF-conditioned medium (CM) or re-pooled fractionated (10K or 3K pool) CAF-CM in the presence of 1 μM doxorubicin.

**Figure S7. Levels of GSSG in LNCaP cells and CAF-CM. A,** GSSG levels in LNCaP cells cultured in fresh (non-CM) or CAF-conditioned medium (CAF-CM), as determined by the glutathione assay described in Methods (mean and s.e.m.; N=3). **B,** Levels of GSH and L-cysteine in RPMI 1640 medium, CAF- and LNCaP-conditioned media, before and after TCEP treatment, as determined by HPLC. GSH and L-cysteine after TCEP treatment indicate levels of GSSG and cystine. **C,** Cystine concentrations in different media assessed by HPLC (mean and s.e.m.; N=2).

**Figure S8. ROS induction by taxol and TBHP treatment.**

**A,** ROS levels in LNCaP cells after 3 hour treatment with 20 nM taxol as assessed by CellROX and expressed as geometric mean fluorescence intensity (mean and s.e.m.; N=3; **p < 0.01). Cells were cultured in fresh (non-CM), CAF-conditioned medium (CAF-CM) or LNCaP-conditioned medium (LNCaP-CM). **B,** ROS levels in LNCaP cells after 1 hour of 200 μM TBHP treatment, or unstained cells in the presence or absence of doxorubicin or taxol (mean and s.e.m.; N=3; ***p < 0.0001).

**Supplementary Table Legend**

**Table S1. Glutathione precursors attenuate doxorubicin accumulation in LNCaP cells.** Reduction in doxorubicin content in LNCaP cells after exposure to the indicated concentrations of GSH, NAC, cysteine or cystine daily for 3 days and 1 μM doxorubicin for 8 hours, in relation to doxorubicin-treated control. Intracellular doxorubicin was determined by FACS and presented as percentage of reduction in doxorubicin content.
